# Comparison of Various Databases for Estimation of Dietary Polyphenol Intake in the Population of Polish Adults

**DOI:** 10.3390/nu7115464

**Published:** 2015-11-11

**Authors:** Anna M. Witkowska, Małgorzata E. Zujko, Anna Waśkiewicz, Katarzyna M. Terlikowska, Walerian Piotrowski

**Affiliations:** 1 Department of Food Commodities Science and Technology, Medical University, Szpitalna 37, Bialystok 15-295, Poland; malgorzata.zujko@umb.edu.pl (M.E.Z.); katarzyna.terlikowska@umb.edu.pl (K.M.T.); 2 Department of Epidemiology, Cardiovascular Disease Prevention and Health Promotion, Institute of Cardiology, Niemodlińska 33, Warsaw 04-635, Poland; awaskiewicz@ikard.pl (A.W.); wpiotrowski@ikard.pl (W.P.)

**Keywords:** polyphenols, flavonoids, databases, intake, adults

## Abstract

The primary aim of the study was to estimate the consumption of polyphenols in a population of 6661 subjects aged between 20 and 74 years representing a cross-section of the Polish society, and the second objective was to compare the intakes of flavonoids calculated on the basis of the two commonly used databases. Daily food consumption data were collected in 2003–2005 using a single 24-hour dietary recall. Intake of total polyphenols was estimated using an online Phenol-Explorer database, and flavonoid intake was determined using following data sources: the United States Department of Agriculture (USDA) database combined of flavonoid and isoflavone databases, and the Phenol-Explorer database. Total polyphenol intake, which was calculated with the Phenol-Explorer database, was 989 mg/day with the major contributions of phenolic acids 556 mg/day and flavonoids 403.5 mg/day. The flavonoid intake calculated on the basis of the USDA databases was 525 mg/day. This study found that tea is the primary source of polyphenols and flavonoids for the studied population, including mainly flavanols, while coffee is the most important contributor of phenolic acids, mostly hydroxycinnamic acids. Our study also demonstrated that flavonoid intakes estimated according to various databases may substantially differ. Further work should be undertaken to expand polyphenol databases to better reflect their food contents.

## 1. Introduction

A number of studies indicate plant foods as particularly beneficial for the prevention of heart disease [1]. Plant-based foods provide multiple bioactive components including vitamin C, E, and polyphenols, which may reduce the risk of cardiovascular diseases. A large group of these compounds, polyphenols, deserve a special attention. Polyphenols are ubiquitous secondary plant metabolites that share common molecular structure of phenol rings, which may be attached to each other or via linkages in various configurations [2]. They are members of four compound classes: flavonoids, phenolic acids, stilbenes and lignans [1]. To date, more than 500 dietary polyphenols have been described in the literature [3]. The two most prominent classes of polyphenols are flavonoids and phenolic acids. Structurally, flavonoids possess at least two phenol rings. On the basis of differences in molecular conformation, six subclasses of compounds can be distinguished: anthocyanins, flavones, flavanones, flavonols, flavanols, and isoflavones [2]. Flavonoids are characterized by antioxidant, antitumor and antiproliferative activities [4]. The predominant dietary sources of flavonoids are tea and fruits. Until now, there have not been specified dietary requirements regarding flavonoid intakes in humans [5]. Phenolic acids, which are widely found in nature, are hydroxy derivatives of the two major compounds: cinnamic and benzoic acids [6]. Hydroxycinnamic acids are known as potent chain-breaking antioxidants, which act by scavenging free radicals via donating hydrogen atoms and electrons [7]. Hydroxycinnamates are present in considerable amounts in such foods as coffee, tea, fruits, vegetables, and whole grains [7].

There are several databases of polyphenol contents in foods. The most commonly used polyphenol data sources are USDA databases of predominant flavonoids [8,9] and an online Phenol-Explorer database of different polyphenols [10,11]. Both data sources are systematically extended to reflect most accurately phenolic contents in food. Use of the two databases, however, has some limitations, that allow only for the estimation of dietary polyphenols, but do not give the definitive values. For that reason, a comparison of the results obtained on the basis of the various data sources may differ.

Polyphenol intake among populations is variable and depends mostly on the food preferences of individual populations. Numerous studies have shown that it can vary within quite a broad range [12,13]. Intake of flavonoids and polyphenols in the general Polish population had not yet been evaluated on the basis of the previously mentioned databases. Therefore, the primary aim of the study was to estimate the consumption of polyphenols in a population representing a cross-section of the Polish society, and the second objective was to compare the intakes of flavonoids calculated on the basis of these two commonly used databases.

## 2. Materials and Methods

### 2.1. Participants

**Figure 1:**
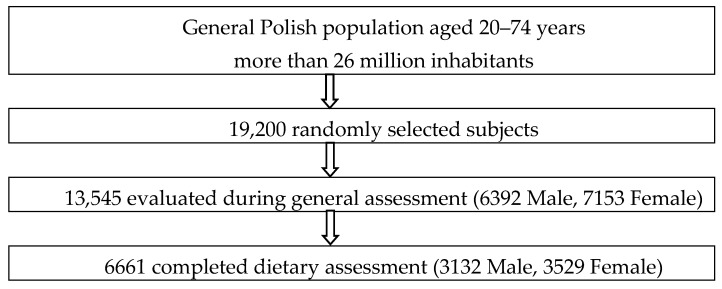
Flow-chart of study participants.

The Polish National Multicenter Health Survey (WOBASZ) is a cross-sectional study representative for the general Polish adult population aged between 20 and 74 years. This study was carried out by the National Institute of Cardiology, Warsaw, Poland, in the years 2003–2005, in collaboration with five Polish medical universities. The response rate was 76.8%. The general assessment involved a sample of more than 13,500 participants (Figure 1). Among this group approximately 50% randomly selected subjects took part in the dietary assessment. Written informed consent was obtained from all participants. The WOBASZ study obtained approval from the Bioethics Committee of the National Institute of Cardiology (no. 708). The rationale, design and methods of the WOBASZ study were described in detail elsewhere [14,15]. Description of selection procedure was introduced in our previous paper [16].

A standardized questionnaire was designed to collect such data as social, demographic, and economic status, physical activity, smoking habit, commune size, marital status, level of education, and net household *per-capita* income. Height and body mass measurements were taken by the personnel trained in the standard procedures. The body mass index (BMI) was calculated from weight in kilograms divided by the square of height in meters. 

Blood pressure (BP) was measured three times on the right arm after 5 min of resting in a sitting position in one-minute intervals and the final BP was provided as an average of the 2nd and 3rd measurements. General description of the study group is given in Table 1.

**Table 1:** General description of the studied population.

Number of Participants (Gender)	*n* = 6661 (3132 Male, 3529 Female)
Age (year) (mean ± SD)	45.2 ± 15.1
BMI (kg/m^2^) (mean ± SD)	26.4 ± 5.2
Systolic Blood Pressure (mmHg) (mean ± SD)	133.6 ± 20.9
Diastolic Blood Pressure (mmHg) (mean ± SD)	82.5 ± 11.9
Smoking (%) ^1^	31.7
Age groups (%)	
20–40 years	37.8
41–60 years	43.9
61–74 years	18.3
Physical activity (%) ^2^	
Low level	35.7
Middle level	16.0
High level	48.3
Commune size (%)	
<8,000 inhabitants	36.7
8,000–40,000 inhabitants	35.5
>40,000 inhabitants	27.8
Marital status (%) ^3^	
Married	71.2
Single	28.8
Level of education (%) ^4^	
Under middle	55.8
Middle	34.0
High	10.2
Household per capita income (%) ^5^	
Low	90.3
Middle	6.9
High	2.8
Energy intake (kcal/day) (mean ± SD)	2061 ± 949

^1^ smoking at least one cigarette a day; ^2^ physical activity at leisure: low level—no such physical activity, for example jogging, cycling, swimming, gardening for at least 30 min a day; middle level—physical activity once a week or less; high level—everyday, almost every day or every second or third day; ^3^ singles: widows/widowers, unmarried, divorced, in separation; ^4^ education level: under middle—no education, partial or completed education for primary level, vocational lower secondary education, partial secondary education; middle—secondary education, partial academic education; high—tertiary education; ^5^ calculated on the basis of the income reported by the study participants. These calculations were taken only those individuals who reported their income.

### 2.2. Dietary Assessment

Daily food consumption data were collected by trained interviewers using a single 24-hour dietary recall. On the basis of the dietary recalls completed by 6661 subjects, it was found that 125 food items and beverages consumed by the participants were sources of polyphenol intakes. These products were grouped in 9 food categories: alcoholic beverages, cereals, cocoa products, fruit, legumes, non-alcoholic beverages, nuts and seeds, oils, and vegetables (listed in alphabetical order). Complex dishes have been split into individual components. These components in which the polyphenols are present, have been taken into consideration. For example, refined wheat flour, which is a component used in typical Polish dishes such as noodles or dumplings, was separated using recipes of dishes contained in Polish Food Composition Tables [17]. These recipes give the amounts of food products required for 100 g dish portion, with consideration of yield factors. For the calculation of polyphenol/flavonoid contents by different databases, the same food items and the same culinary techniques were taken into account.

### 2.3. Estimation of Dietary Polyphenol Intakes

Intake of total polyphenols was estimated using an online Phenol-Explorer database [10], which contains mean representative content values for 502 individual polyphenols belonging to four polyphenol classes (flavonoids, phenolic acids, lignans, and stilbenes) and other polyphenols (including, among others, tyrosols) in 452 food products [11]. Flavonoids included anthocyanins, chalcones, dihydrochalcones, dihydroflavonols, flavanols, flavanones, flavones, flavonols, and isoflavones (listed in alphabetical order); phenolic acids included hydroxybenzoic acids, hydroxycinnamic acids, and hydroxyphenacetic acids; and the remaining polyphenols included lignans, stilbenes, others.

The high performance liquid chromatography (HPLC) data were used to calculate the results. For the analysis of lignans, which cannot be released with normal extraction conditions, the data obtained with HPLC method, which applies acid hydrolysis, were used.

Dietary flavonoid intake was determined using following data sources: the USDA database combined of flavonoid [8] and isoflavone [9] databases, and the Phenol-Explorer database. The USDA Database for the Flavonoid Content of Selected Foods, release 3.1 (May 2014), reports 26 predominant flavonoids in 500 food items, and the USDA Database For Isoflavone Content Of Selected Food Items, release 2.0 (September 2008) contains data for the total as well for 6 specified isoflavones (daidzein, genistein, glycitein, coumestrol, biochanin A, and formononetin) in 557 food items [9].

The meal preparation techniques were taken into consideration as factors influencing polyphenol contents in food items. The USDA databases show data both for raw and processed food items taking into account different preparation techniques. For the data obtained with Phenol-Explorer, the retention factors (RFs) were used to convert polyphenol contents in raw foods to the contents found in the processed foods. The content of polyphenols in the raw food was multiplied by appropriate RF specific for such culinary techniques as boiling, frying, or baking. These polyphenol RFs are implemented in the Phenol-Explorer database. 

Eventually, polyphenol/flavonoid daily intakes were determined by multiplying the daily consumption of individual food items by polyphenol/flavonoid contents in these food items. The data for individual classes of polyphenols and the subclasses of flavonoids are expressed as aglycone equivalents.

### 2.4. Data Analysis

Data analyses were processed using Statistical Analysis System (SAS, version 9.2, SAS Institute Inc., Cary, NC, USA). Results were analyzed using the PROC GLM procedure. Analysis of covariance (ANCOVA) adjusted for season was used to determine LSMEANS for nutritional factors. The variable “season” was introduced to the model as a confounding factor to eliminate its impact on food consumption.

Dietary polyphenol and flavonoid intakes were expressed as means and standard deviations (± SD). Contributions of individual classes of polyphenols, including subclasses of flavonoids, to the total polyphenol intake and the contributions of food categories and individual food items to intakes of particular flavonoids and polyphenols were presented as percentages. To compare mean values of the analyzed factors a paired *t*-Student’s test was used. *p* values less than 0.05 were considered statistically significant.

## 3. Results

Total polyphenol intake that was calculated with the Phenol-Explorer database for the studied cross-section of the Polish society was calculated as aglycone equivalents, which amounted to 989 mg/day with the highest contribution of phenolic acids 556 mg/day (56%) and flavonoids 403.5 mg/day (~41%) (Table 2). The consumption of lignans, stilbenes and other polyphenols was minor 29.5 mg/day (3% of total polyphenols). The flavonoid intake calculated on the basis of the USDA databases [8,9] amounted to 525 mg/day and has been found 30% higher as compared to the data on flavonoids estimated using Phenol-Explorer (*p* value less than 0.001). The USDA databases do not contain data on phenolic acids, lignans, stilbenes and other polyphenols.

**Table 2:** Polyphenol intake (mg/day) in the studied population (*n* = 6661).

	USDA Flavonoid Database *		Phenol-Explorer	
	mg/day (mean ± SD)	% Contribution to total polyphenol intake	mg/day (mean ± SD)	% Contribution to total polyphenol intake
Flavonoids	524.6 ± 155 **	unknown	403.5 ± 150 **	40.8
Phenolic acids	-	-	556.3 ± 204	56.2
Lignans, stilbenes, other polyphenols	-	-	29.5 ± 9	3.0
TOTAL polyphenol intake	-	-	989.3 ± 360	100

* From the USDA databases of flavonoids and isoflavones [8,9]; ** statistically significant differences at *p* < 0.001.

According to Phenol-Explorer, non-alcoholic beverages were main dietary sources of total polyphenols 743.68 mg/day (75% of total polyphenols) including phenolic acids 422.13 mg/day (76% of phenolic acids) (Table 3). Moreover, according to both data sources, Phenol-Explorer and the USDA database, non-alcoholic beverages were also main dietary sources of flavonoids 316.91 mg/day (78.5% of flavonoids) and 471.00 mg/day (90% of flavonoids), respectively. Important polyphenol, flavonoid (according to both databases) and phenolic acid sources were also fruits and vegetables. Major discrepancies between the flavonoid intakes established with the USDA databases and Phenol-Explorer were found for almost all food groups (*p* values less than 0.001). The greatest differences were observed in the case of cereal products. The flavonoid intake from cereals, calculated with the Phenol-Explorer, exceeded more than 30 times this calculated using the USDA databases.

**Table 3:** Contributions of food categories to flavonoid, phenolic acid and total polyphenol intakes (mg/day) (*n* = 6661).

Food Categories	Flavonoids (mg/day) (mean ± SD)		Phenolic Acids (mg/day) (mean ± SD)		Total Polyphenols (mg/day) (mean ± SD)	
	USDA *	Phenol-Explorer	USDA	Phenol-Explorer	USDA	Phenol-Explorer
Alcoholic beverages	1.00 ± 1.46 **	1.24 ± 3.10 **	-	1.41 ± 1.43	-	2.92 ± 2.60
Cereals	0.11 ± 0.13 **	3.84 ± 2.22 **	-	2.44 ± 2.43	-	17.66 ± 8.24
Cocoa products	0.67 ± 3.51 **	2.69 ± 8.05 **	-	0.17 ± 0.91	-	2.87 ± 6.22
Fruit	39.09 ± 35.49 **	74.82 ± 56.56 **	-	39.45 ± 75.00	-	114.72 ± 54.26
Legumes	0.20 ± 1.93 **	1.53 ± 2.27 **	-	0.06 ± 0.15	-	1.59 ± 1.76
Non-alcoholic beverages	471.00 ± 128.42 **	316.91 ± 70.42 **	-	422.13 ± 229.95	-	743.68 ± 116.40
Nuts and seeds	0.01 ± 0.13	0.01 ± 0.10	-	1.37 ± 10.12	-	1.39 ± 4.97
Oils	0.12 ± 0.10	0 ± 0	-	0.17 ± 0.07	-	1.21 ± 0.50
Vegetables	12.53 ± 6.30 **	2.31 ± 5.19 **	-	89.12 ± 49.52	-	103.24 ± 24.47

* From the USDA databases of flavonoids and isoflavones [8,9]; ** statistically significant differences at *p* < 0.001.

Though Phenol-Explorer gives the higher results for alcoholic beverages, cereals, cocoa products, fruit and legumes, the flavonoid content of non-alcoholic beverages (mostly tea) and of vegetables, provided by the USDA databases, cause that total flavonoid intake assessed with the USDA databases is higher than this estimated with Phenol-Explorer.

According to the USDA flavonoid databases, flavanols were predominant dietary flavonoid sources 461.79 mg/day (88% of total flavonoids) for the studied population, followed by flavonols 31.95 mg/day (6.1%) and anthocyanins 20.93 mg/day (4.0%) (Table 4). Other flavonoids such as flavanones, flavones and isoflavones provided 9.95 mg/day (1.85%). Tea consumption accounted for more than 96% of flavanol intake. The major source of flavonols, ~50%, was tea, whereas for anthocyanins they were plums at about 30%. Generally, the main sources of total flavonoids for the studied population were: tea (87.1%), apples (2.97%), plums (1.18%) and orange juice (1.01%).

**Table 4:** Dietary flavonoid intakes calculated with the USDA database * and their major food contributors (*n* = 6661).

Flavonoid Class	Flavonoid Intake mg/day (mean ± SD)	% of Total Flavonoids	Major Food Contributors to Flavonoid Intakes from Individual Flavonoid Subclasses (%)
Anthocyanins	20.93 ± 31.37	4.0	plums (29.5), blackcurrant juice (15.3), strawberries (14.6), red currant (10.6)
Flavanols	461.79 ± 108.15	88.0	tea (96.4), apples (2.3)
Flavanones	9.16 ± 15.50	1.7	orange juice (57.6), oranges (14.3), grapefruit juice (6.8)
Flavones	0.63 ± 0.51	0.12	celeriac (24.0), apples (22.0), Savoy cabbage (11.0), green pepper (8.4)
Flavonols	31.95 ± 5.95	6.1	tea (47.1), apples (15.1), onion (11.4), potatoes (10.8)
Isoflavones	0.16 ± 0.88	0.03	coffee (41.2), soy flour (16.8), soybeans (16.2)

* From the USDA databases of flavonoids and isoflavones [8,9].

In Table 5, polyphenol intakes and their main dietary sources are listed with regard to particular classes and subclasses of polyphenols. Of the flavonoids, quantitatively, the largest groups were flavanols, 314.68 mg/day (78% of flavonoids), followed by flavonols, 47.91 mg/day (11.9%), and anthocyanins, 22.03 mg/day (5.5%). Other flavonoids provided 18.73 mg/day (4.6%). When it comes to the sources of listed subclasses of flavonoids, tea was predominant source of flavanols (86.3%) and flavonols (72.2%), while strawberries (37.6%), plums (23.9%) and sweet cherries (16.2%) were the main sources of anthocyanins. Quantitatively, the largest sources of total flavonoids for the study group were: tea (75.9%), apples (10.5%), plums (2.6%), and strawberries (2.1%).

Another group of polyphenols, phenolic acids, were most widely represented by hydroxycinnamic acids 492.05 mg/day (88.5% of phenolic acids) and subsequently by hydroxybenzoic acids 64.22 mg/day (11.5%). Coffee was the major source of hydroxycinnamic acids (71%), and tea was the main source of hydroxybenzoic acids (93.5%).

In the case of the other groups of phenolic compounds, lignans amounted to 12.09 mg/day (41.1% of lignans, stilbenes and other polyphenols), stilbenes to 0.18 mg/day (0.6%), and the other polyphenols to 17.14 mg/day (58.3%). The main dietary sources of lignans were cucumbers (41.2%) and red cabbage (22.0%), and the main sources of stilbenes were red currants (approximately 26%), red wine (24%), strawberries (22%) and white wine (16%). Dietary sources of other polyphenols were mostly whole meal rye bread (60%) and coffee (24%).

## 4. Discussion

As it was shown by the studies carried out in various research centers, the consumption of polyphenols may differ among populations, especially when values derived with different analytical methods are compared. Total polyphenol intake 989 mg/day (expressed as aglycone equivalents) in the current study was less than this assessed with the total polyphenol assay. As we have shown in a previous study, the intake of polyphenols for this particular test group was 1031–1172 mg GAE (Gallic acid equivalents)/day, and was calculated on the basis of an own database of polyphenols, which were assessed with the Folin–Ciocalteu (FC) method [18]. As we expected, these discrepancies might have resulted mainly from the application of two different analytical methods of polyphenol determination (FC method *vs.* high pressure liquid chromatography (HPLC) method) as well as from minor shortcomings of polyphenol contents for particular food items in the searched databases. On the other hand, the FC method can give higher results due to the presence in food of non-phenolic substances, which can react with the FC reagent (FCR), including some vitamins, thiols and amino acids [19]. Therefore the FC method can only be considered as a rough measure of polyphenols for most foods [19]. Major disadvantage of the FC method is quantitative analysis only, but it does not give answers to the question what is the composition of polyphenols. In the present work, in addition to quantitative analysis, qualitative analysis was made, both of which used common databases.

**Table 5:** Dietary polyphenol intakes calculated with the Phenol-Explorer database and their major food contributors (*n* = 6661).

Flavonoids			
	**Phenol-Explorer mg/day (mean ± SD)**	**% of Total Flavonoids**	**Major Food Contributors to Polyphenol Intakes from Individual Polyphenol Subgroups (%)**
Anthocyanins	22.03 ± 7.26	5.5	strawberries (37.6), plums (23.9), sweet cherries (16.2)
Chalcones	0.0006 ± 0.0003	trace	beer (100)
Dihydrochalcones	7.02 ± 2.78	1.7	apples (89.0), apple juice (11.0)
Dihydroflavonols	0.07 ± 0.03	0.017	red wine (75.7), white wine (21.7)
Flavanols	314.68 ± 123.14	78.0	tea (86.3), apples (8.9), plums (1.6)
Flavanones	7.28 ± 2.67	1.8	orange juice (55.7), oranges (19.0), grapefruit juice (18.8)
Flavones	4.32 ± 1.61	1.1	wheat flour (79.0), orange juice (14.2), celeriac (3.1)
Flavonols	47.91 ± 17.27	11.9	tea (72.2), apples (16.7), beans (3.1)
Isoflavones	0.03 ± 0.01	0.008	soy flour (46.4), soybeans (43.9)
**Phenolic Acids**			
	**Phenol-Explorer mg/day (mean ± SD)**	**% of Phenolic Acids**	**Major Food Contributors to Polyphenol Intakes from Individual Polyphenol Subgroups (%)**
Hydroxybenzoic acids	64.22 ± 26.01	11.5	tea (93.5), apples (2.0)
Hydroxycinnamic acids	492.05 ± 178.15	88.5	coffee (70.6), potatoes (16.7), apples (4.2)
Hydroxyphenacetic acids	0.04 ± 0.02	trace	beer (89.2), white wine (6.5), red wine (4.2)
**Lignans, Stilbenes, Other Polyphenols**			
	**Phenol-Explorer mg/day (mean ± SD)**	**% of Lignans, Stilbenes, Other Polyphenols**	**Major Food Contributors to Polyphenol Intakes from Individual Polyphenol Subgroups (%)**
Lignans	12.09 ± 4.98	41.1	cucumber (41.2), red cabbage (22.0), tangerines (9.7), brussels sprouts (6.7)
Stilbenes	0.18 ± 0.06	0.6	red currant (25.6), red wine (24.1), strawberries (21.9), white wine (16.1)
Others	17.14 ± 6.01	58.3	wholemeal rye bread (60.1), coffee (24.4), canola oil (6.0)

Substantially, the 989 mg/day polyphenol intake estimated in this study for the Polish population was higher compared to polyphenol intakes estimated for the population of Greece, 744 mg/day in men and 584 mg/day in women [12], population of high cardiovascular risk in Spain 820 mg/day [20] and Finland 863 mg/day [21], but lower than the polyphenol intake in France at 1193 mg/day [13] and in a Spanish population consuming the Mediterranean diet (1173 mg/day) [22]. As we have shown earlier, the consumption of phenolics may vary due to age and gender [18,23]. In our present study, the age range of the studied population was 20–74 years, while, for example, in French studies it was 45–60 years [13]. It is characteristic that older people tend to have reduced intakes of polyphenols compared with the younger age groups [23,24]. The intake of polyphenols in our study largely corresponds to various age groups of Poland’s adult society, and involves elderly participants, which could be one of the supposed reasons for the reduced intake of polyphenols by the entire studied population.

A further consideration was the appropriate selection of the study groups taken for comparison. As some studies show, polyphenol intakes in general populations may differ from the intakes by communities [12]. This finding can be confirmed by our research. If we compare our data with other studies carried out regionally in Poland, we find that they may slightly vary. In the population of the city of Krakow, Poland, phenolic intake was 1092 mg/day (calculated as aglycones) [24]. With the sum of polyphenols of 989 mg/day, our results are just about 10% lower than in this urban population.

Flavonoids are a large class of polyphenols. Both data sources used in this study, the USDA databases and Phenol-Explorer, give the possibility to estimate flavonoid contents in the diet. The USDA tables provide data for six subclasses of flavonoids: anthocyanins, flavanols, flavanones, flavones, flavonols [8] and isoflavones [9]. In turn, Phenol-Explorer, in addition to the listed, provides data for the precursor flavonoid compounds, which are chalcones, dihydrochalcones and dihydroflavonols. We found that the intake of flavonoids by the study participants was about one third higher when calculated using the USDA databases in relation to the Phenol-Explorer database, 525 mg/day *vs.* 404 mg/day. The main reason for these discrepancies might be better characterization of tea flavonoids by the USDA database. However, Phenol-Explorer data of polyphenol contents are more complete regarding cereal products. Compared to other research, the results obtained on the basis of Phenol-Explorer were about one third lower than these of the urban Polish population 572 mg/day (as aglycone equivalents), for which the results were calculated using the same method [24] and similar to the Spanish PREDIMED cohort 443 mg/day [20].

In our study, the latest release of the USDA flavonoid database (2014) of five subclasses of flavonoids (anthocyanins, flavanols, flavanones, flavones, and flavonols) was used, which could affect the results of comparisons with populations in other studies, which used the earlier versions of the USDA flavonoid database. For example, in the European Prospective Investigation into Cancer and Nutrition (EPIC) study carried out in 2010 in Spain, the intake of flavonoids by the adult population aged 35–64 years amounted to 313 mg/day [25]. Compared to this research, flavonoid intake in our study was more than 40% higher. However, it should be noted that the USDA flavonoid database used in the EPIC study was from 2007. Since then, it has been significantly upgraded with many new foods and new data concerning flavonoid contents. By comparing the sources of flavonoids for various populations, it can be concluded that they may differ depending on the preferences for specific types of food. In our study and in the US diet, they were mainly tea, 75.9% and 82.8% [26], respectively, while in Spain they were oranges, apples, and red wine [20].

In the case of the studied Polish population, intake of phenolic acids exceeds the intake of flavonoids, mainly due to the consumption of coffee, tea, potatoes and apples. Earlier studies found that phenolic acids are the main contributors to the total polyphenol intake in the non-Mediterranean countries, and they accounted for 57% and 53% polyphenol contribution depending on gender [12]. We observed a similar 56% phenolic acid contribution. As in the Spanish Prevención con Dieta Mediterránea (PREDIMED) cohort study [20], hydroxycinnamic acids were main contributors to the total polyphenol intake in our study. However, they were only slightly exceeded by the intake of flavonoids from tea in our study.

In addition to flavonoids and phenolic acids, one source of polyphenols for the studied population was lignans. Cabbage and fruit vegetables are listed as primary sources of lignans for the European populations [27], which is also confirmed by the results of our research. Another minor phenolic compounds assessed in this study was stilbenes. These phenolics are particularly abundant in red wine, especially in the form of stilbenoid resveratrol. In the current research, red currant, red wine and strawberries almost equally contributed to the intake of stilbenes, from ~22 to ~26% of the total stilbenes. In contrast to the intakes of lignans and stilbenes in other European regions, from 3.1 to 9.1 mg/day [12], these intakes in the studied Polish population were much greater and accounted to 12.3 mg/day, mainly due to the consumption of cucumbers and red cabbage, which are popular culinary vegetables in Poland.

According to Phenol-Explorer, non-alcoholic beverages accounted for 75% of dietary intake of polyphenols by the studied representative sample of the Polish population, and the tea for 76% intake of flavonoids, while coffee for 62% intake of phenolic acids. In addition, tea provided 37.0% polyphenols, and the coffee 35.5%. Such results with regard to tea are not surprising, because Poland is a country of high tea consumption. With 1 kg per capita intake, Poland ranks fourth in Europe for tea consumption, and ninth worldwide [28]. As it was found in the studies involving populations of Mediterranean and non-Mediterranean countries, high contribution of coffee and tea to the polyphenol intake is typical for the non-Mediterranean countries [12]. However, in contrast to other non-Mediterranean countries [12], contribution of polyphenols from tea in our study was considerable, 37.0% *vs.* 17.0% in other countries.

## 5. Conclusions

To the best of our knowledge this is the first attempt to estimate both the intake of polyphenols, including flavonoids, using the Phenol-Explorer database and the intake of flavonoids assessed with the latest release of the USDA database, in the representative sample of Poland’s general population. This study found that tea is the primary source of polyphenols and flavonoids for the studied population, including mainly flavanols, while coffee is the most important contributor of phenolic acids, mostly hydroxycinnamic acids. Our study also demonstrated that flavonoid intakes estimated according to various databases might substantially differ. If there is a need to compare intakes of flavonoids between populations, the use of one database should be preferred. Presumably, the use of several combined databases can truly reflect the intake of flavonoids, but it will be difficult to make comparisons between populations for which only one method of calculation has been used. Therefore, further work should be undertaken to expand polyphenol databases to better reflect their food contents.
